# A Rare Colocalization of Lichen Planus and Vitiligo

**DOI:** 10.1155/2015/840193

**Published:** 2015-11-24

**Authors:** David Veitch, Georgios Kravvas, Sian Hughes, Christopher Bunker

**Affiliations:** ^1^Department of Dermatology, University College London Hospitals, London NW1 2BU, UK; ^2^Department of Histopathology, University College London Hospitals, London NW1 2BU, UK

## Abstract

We report an unusual manifestation of vitiligo colocalizing with lichen planus (LP). A 76-year-old Greek male presented with a history of a red, scaly, itchy, asymmetrical patch located at the umbilicus within a well-demarcated depigmented macule of vitiligo. Histology showed features of a lichenoid interface dermatitis, favouring a diagnosis of LP. Colocalization of LP and vitiligo has rarely been reported in the literature. After reviewing the literature, we believe that at present there is insufficient evidence to resolve the uncertainties in the aetiology of this colocalization. It seems to us that the association between LP and vitiligo is more than coincidental, but none of the theories discussed in this paper can sufficiently account for it. Rather, the association is likely to be multifactorial in its pathogenesis.

## 1. Introduction

Colocalization of lichen planus (LP) and vitiligo has rarely been reported in the literature. A number of different patterns of association have been recognised.

The fact that both LP and vitiligo are common, each said to affect 1-2% of the general population, may mean that their association is merely a coincidence and may not represent any mutual interrelationship [1, 2]. On the other hand, a number of publications suggest that a causal link must be present since similar immunological mechanisms are shared by both conditions [3–6]. The aetiology of neither LP nor vitiligo is known. We discuss the theories below.

## 2. Case Report

**Figure 1:**
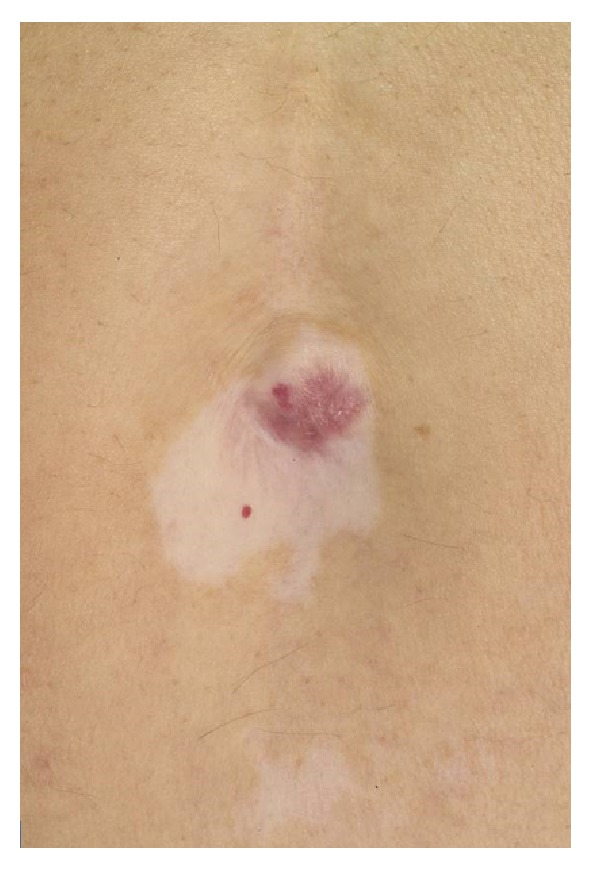
Umbilicus demonstrating vitiligo with overlying lichen planus.

**Figure 2:**
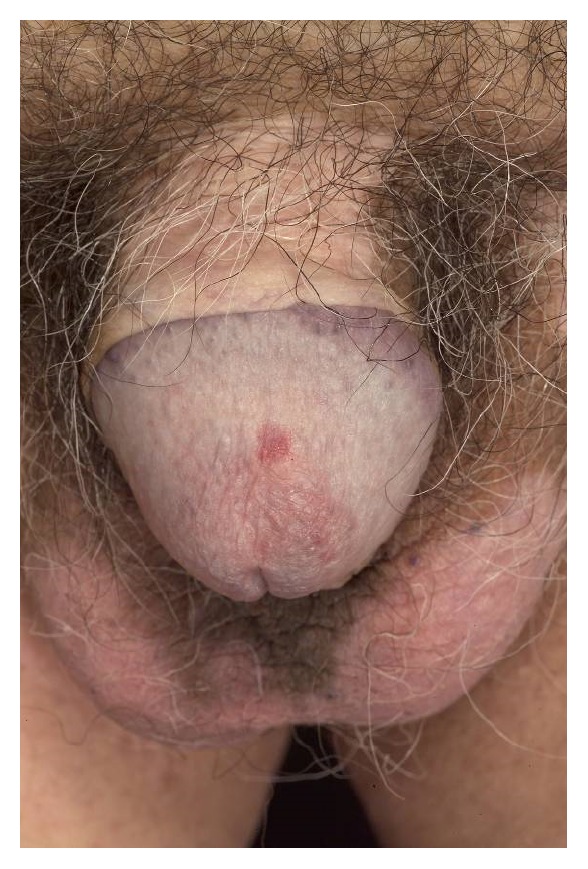
Scrotal and penile vitiligo with a region of lichen planus lesion on the glans.

A 76-year-old Greek male (retired plumber) presented with a 5-6-year history of a red, scaly, itchy, asymmetrical patch (more prominent after sun exposure, he averred) located at the umbilicus within a well-demarcated depigmented macule of vitiligo: he gave a 30-year history of generalized vitiligo affecting the genitals, umbilicus, axillae, and hands (Figure 1). The vitiligo was inactive, stable, and nonprogressive. He also had a solitary red, itchy papule of the glans penis present for over 3 years (Figure 2). Hair, scalp, nails, and mucosae were otherwise completely normal.

His medical background included ischaemic heart disease, paroxysmal atrial fibrillation (with multiple failed ablations), moderate aortic stenosis, obstructive sleep apnoea, bleeding duodenal ulcer (emergency laparotomy), benign prostatic hypertrophy, and thalassaemia trait. There was no history of autoimmune disease in the patient or his family. His medication consisted of atorvastatin, spironolactone, losartan, lansoprazole, finasteride, and warfarin. He was not on topical or systemic therapy for vitiligo due to the longstanding, stable nature of the disease and relative lack of psychosocial morbidity. He was given sun protection advice.

**Figure 3:**
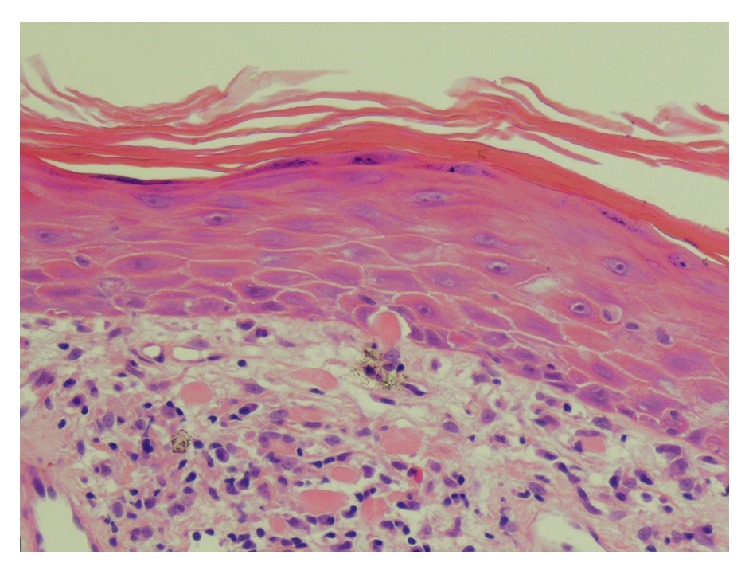
Umbilical 4 mm punch biopsy magnification ×200, Hematoxylin and Eosin stain. Features described in text.

Bowen's disease was suspected and a 4 mm punch biopsy of the lesion on the umbilicus was performed. Histology showed hyperkeratosis and cytoid bodies: Civatte (epidermal) and colloid (dermal). There was some epidermal flattening suggestive of resolving lichen planus. There was a band-like inflammatory cell infiltrate composed of lymphocytes, histiocytes, and occasional eosinophils (Figure 3). The features were those of a lichenoid interface dermatitis, favouring a diagnosis of lichen planus.

He was prescribed clobetasol propionate ointment which he applied once daily for 4 weeks to both the umbilicus and glans. Both lesions completely resolved leaving only mild telangiectatic change over the umbilicus.

## 3. Discussion

A number of associations between LP and vitiligo have been reported.

LP lesions have been described as confined to vitiliginous areas alone or even affecting both normal and vitiliginous skin [3, 4]. They have been said to be more severe on sun-exposed vitiliginous areas, less so on sun-exposed normally pigmented skin, and the least severe on covered areas [7]. In most cases vitiligo is described as the precursor disease but concomitant onset and progression of both conditions has also been noted [1].

Various theories for the aetiology of vitiligo have been advanced and the autoimmune hypothesis is the prevailing view. This is because of the association of vitiligo with other autoimmune disorders, the higher frequency of organ specific antibodies found in patients with vitiligo compared with the general public, and the detection of melanocyte-specific antibodies detected in patients with vitiligo [8].

LP also occurs in patients with autoimmune diseases other than vitiligo. Within lesions CD4 and CD8 cells accumulate in the dermis where they cause lysis of keratinocytes. LP is thought to be an immunologically mediated disorder driven by a T cell response to an unknown antigen or antigens [4, 9].

Baghestani et al. have suggested that sun-exposed depigmented areas play an important part in the initiation of LP that then extends to involve normal skin. A popular hypothesis holds that photodamage within areas of vitiligo causes the release of inflammatory mediators, thus promoting the accumulation of effector T cells as are seen in LP. According to this theory, in vitiligo-affected patients, LP is more likely to be encountered in abnormally pigmented skin and in sun-exposed areas [4, 7]. To support this further, it has been well documented that psoralen and UVA- (PUVA-) induced lichenoid changes can occur in vitiliginous skin [2, 4].

In contrast to the actinic damage theory, there have been descriptions of cases in which LP is confined solely on nonexposed areas of the skin, such as the scrotum, inguinal folds, and thighs [4].

Another theory is based on the evident manifestation of the Koebner phenomenon in LP; koebnerisation describes the eruption of an inflammatory skin disease following mechanical injury of the skin [10]. It has been proposed that cellular injury in vitiligo-affected skin, augmented by the effects of solar damage, modifies the mechanisms responsible for the Koebner phenomenon, resulting in LP on sun-exposed skin [1]. However, the presentation in our patient may represent Wolf's isotopic response, a subtype of the Koebner phenomenon, in which one skin disease may trigger a second, pathogenically unrelated skin lesion [10].

Yet another conjecture is that long-standing vitiligo alters the expression of antigens identified by effector T cells in LP or inactivates suppressor T cells, thus leading to the pathophenotype of LP [4].

Göktay et al. have suggested that tumor necrosis factor (TNF) may be the crucial mediator in the colocalization of LP and vitiligo. The rationale is that TNF-*α* immunoreactivity has often been detected in oral and cutaneous LP and that enhanced levels of TNF-*α* production from melanocytes have also been detected in patients with vitiligo [4].

But the literature also contains cases of associated LP and vitiligo that are not readily accommodated by these theories. Baran et al. described lesions of LP, in the shape of a rim with sharp borders, affecting only the pigmented skin around several vitiliginous patches [6]. Wayte and Wilkinson reported a case in which widespread LP spared all areas of long-standing vitiligo, with sharp borders separating the two types of lesions. Indeed, Wayte and Wilkinson proposed that changes in vitiliginous skin actually protected against lichenoid transformation [5].

After reviewing the literature, we believe that at present there is insufficient evidence to resolve the uncertainties. It seems to us that the association between LP and vitiligo is more than coincidental, but none of the above theories can sufficiently account for it. Rather, the association is likely to be multifactorial in its pathogenesis.

## References

[1] Hefazi M. T., Mosiehi H., Amir E., Ostradahimi N. (2009). Colocalization of Lichen Planus and vitiligo: challenging the universality of current theories. *Iranian Journal of Dermatology*.

[2] Porter S. R., Scully C., Eveson J. W. (1994). Coexistence of lichen planus and vitiligo is coincidental. *Clinical and Experimental Dermatology*.

[3] Ujiie H., Sawamura D., Shimizu H. (2006). Development of lichen planus and psoriasis on lesions of vitiligo vulgaris. *Clinical and Experimental Dermatology*.

[4] Göktay F., Mansur A. T., Aydingöz I. E. (2006). Colocalization of vitiligo and lichen planus on scrotal skin: a finding contrary to the actinic damage theory. *Dermatology*.

[5] Wayte J., Wilkinson J. D. (1995). Unilateral lichen planus, sparing vitiliginous skin. *British Journal of Dermatology*.

[6] Baran R., Ortonne J. P., Perrin C. (1997). Vitiligo associated with a lichen planus border. *Dermatology*.

[7] Baghestani S., Moosavi A., Eftekhari T. (2013). Familial colocalization of lichen planus and vitiligo on sun exposed areas. *Annals of Dermatology*.

[8] Anstey A. V., Bruns T., Breathnach S., Griffiths C. (2010). Disorders of skin colour. *Rook's Textbook of Dermatology*.

[9] Breathnach S. M., Bruns T., Breathnach S., Griffiths C. (2010). Lichen planus and lichenoid disorders. *Rook's Textbook of Dermatology*.

[10] Kroth J., Tischer J., Samtleben W., Weiss C., Ruzicka T., Wollenberg A. (2011). Isotopic response, Köbner phenomenon and Renbök phenomenon following herpes zoster. *Journal of Dermatology*.

